# New insights in the probability distributions of wave-by-wave overtopping volumes at vertical breakwaters

**DOI:** 10.1038/s41598-022-20464-5

**Published:** 2022-09-28

**Authors:** M. Salauddin, J. J. O’Sullivan, S. Abolfathi, Z. Peng, S. Dong, J. M. Pearson

**Affiliations:** 1 UCD Dooge Centre for Water Resources Research, UCD School of Civil Engineering and UCD Earth Institute, University College Dublin, Dublin, Ireland; 2 School of Engineering, University of Warwick, Coventry, UK; 3 State Key Laboratory of Estuarine and Coastal Research, Institute of Eco-Chongming, East China Normal University, Shanghai, 200241 PR China

**Keywords:** Civil engineering, Hydrology

## Abstract

Advances in the development of prediction tools for wave overtopping allow now for overtopping volumes to be estimated with good accuracy, with the combined use of mean overtopping rates and maximum wave by wave overtopping volumes in a sequence of wave overtopping events. While previous literature has tended to focus on mean overtopping rates at coastal structures, limited studies have investigated the wave by wave overtopping volumes at coastal sea defences; in particular, a paucity of studies have focussed on the prediction of the shape parameter in the Weibull distribution (i.e., Weibull *b*) of overtopping volumes. This study provides new insights on the probability distribution of individual wave overtopping volumes at plain vertical seawalls by analysing the measured Weibull *b* values derived from a series of laboratory experiments on seawalls performed on a wide range of wave conditions and crest freeboards. The influence of wave conditions (wave steepness, significant wave height), structural parameters (crest freeboard, toe water depth), impulsiveness, probability of overtopping waves, and overtopping discharge on Weibull *b* parameter were examined, and then compared with the well-established empirical formulae. For the conditions covered within this study, it was found that the probability distribution of wave-by-wave overtopping volumes follow a 2-parameter Weibull distribution. No apparent differences in Weibull *b* values were reported with the variation of incident wave steepness and impulsiveness parameter. Results of this study revealed that Weibull *b* values at vertical walls, subjected to non-impulsive wave conditions, can be predicted reasonably well using relative freeboard and relative overtopping rates. A new unified formula is proposed for the estimation of Weibull *b* values at vertical walls under impulsive and non-impulsive wave attack.

## Introduction

Coastal protection infrastructure is primarily designed to limit wave induced overtopping hazards and protect people and properties behind the defence lines. However, the combined effects of sea level rise and the greater frequency and intensity of extreme storm surges that are predicted into the future from global climate change^1–3^ are likely to increase the pressures on our critical coastal defences. The capacity to reliably predict overtopping volumes from extreme waves at sea defences, of which vertical seawalls, sloping dikes and rubble mound breakwaters, are common, continues to be a central tenet in assessing coastal risk and developing risk management strategies to mitigate these risks.

Historically, the prediction of overtopping at vertical seawalls^4–10^, sloping dikes^11–13^ and rubble mound breakwaters^11,14–17^, has relied exclusively on estimates of the mean overtopping discharge, *q* (in m^3^/s per metre structure width) behind the crest of a structure. Recent advances however, in the development of robust prediction tools for wave induced overtopping hazards have confirmed that tolerable overtopping limits (defined in terms of the allowable volume of overtopping water) can be better estimated using both mean overtopping discharge, together with the maximum individual overtopping wave volume in a sea state^17,18^. Although a significant number of studies have investigated mean overtopping discharge at sea defences, studies that focus specifically on the distribution of individual wave overtopping volumes in a sequence of overtopping events remain more limited. This is somewhat surprising in that it is generally recognised^19–21^ that consideration of individual overtopping volumes is more critical in profiling the overtopping risk along defence lines.

However, recent years have seen an increase in the body of research that has focussed on the derivation of reliable predictions of maximum individual overtopping volumes, *V*_*max*_, in a test sequence (see for example^20–26^). It is generally accepted that wave-by-wave overtopping volumes in an overtopping sequence adhere to the two-parameter Weibull distribution function (with scale parameter *a* and shape parameter (or slope), *b*)^4,9,10,27,28^. Clearly, a reliable prediction of the Weibull *b* shape parameter (or Weibull *b*) in a distribution of individual overtopping volumes is critical for reliable estimates of maximum individual overtopping volumes^20^. However, no formal guidance exists for determining the shape parameter in these instances and several approaches for fitting shape parameters to Weibull distributions are reported in literature – some research using the upper part of the Weibull distribution function^6,20,25,28–30^, with other research using 10%^23^ and 20%^31^ of the upper part of the distribution.

Although recent studies on wave-by-wave overtopping volumes at rubble mound breakwaters and smooth sloping sea defences have resulted in robust empirical predictions of Weibull *b* as a function of the relative crest freeboard of the structure or as a function of relative overtopping discharge, an appropriate predictive tool for the case of vertical breakwaters has yet to be developed. While Besley^4^ has developed an empirical relationship for estimating Weibull *b* that is applicable to vertical seawalls, this is based only on incident wave steepness, without considering the influence of wave overtopping discharge or relative freeboard on the distribution of Weibull *b* values. Furthermore, while Besley’s relationship has yet to been validated with laboratory measurements for vertical seawalls, it has been found to be unreliable for rubble mound structures in some studies (see for example^13^). Therefore, scope exists for developing more accurate predictions of maximum individual overtopping discharge and distribution of wave-by-wave overtopping volumes at vertical seawalls. Here we report on the influence of a range of wave characteristics on the shape parameter of the Weibull distribution for wave-by-wave overtopping at vertical seawalls. An extensive suite of laboratory tests undertaken in two separate research-grade laboratory wave flumes, and which cover a wide range of wave conditions are included in the research. Weibull *b* values are compared with empirical predictions from existing tools and the performance of these formulae for Weibull *b* prediction at vertical seawalls is assessed. Based on the results, a new formula is proposed for the estimation of Weibull *b* at vertical walls under impulsive wave attack.

The paper is organised in five sections. Section "Overview of Existing Research on Wave-by-Wave Overtopping Volumes" provides a background to the current study by presenting an overview of previously published research on wave-by-wave overtopping volumes on sea defences. In Sect. "Laboratory data", the tested structure, the test conditions and the data collection and analysis are detailed. The analyses of Weibull *b*, in which the effects of wave conditions and structural parameters on Weibull *b* values are systematically explored are outlined in Sect. "Results and discussion" and following this, the statistical performance of both existing tools. and the newly proposed formula, for estimating Weibull *b* at vertical seawalls is presented in Sect. 4. Lastly, Sect. "Conclusions" provides concluding remarks, summarising what are the most reliable empirical tools for estimating Weibull shape parameters on vertical breakwaters.

## Overview of existing research on wave-by-wave overtopping volumes

The distribution of individual wave overtopping volumes in an overtopping sequence follows a two-parameter Weibull distribution function, first introduced by Van der Meer and Janseen^15^ for sloping dikes, and by Franco^10^ for vertical coastal structures. In more recent years, a number of parametric studies have focussed on investigating the two-parameter Weibull distribution for wave-by-wave overtopping volumes at sea defences of varying geometry and characteristics (see for example the works of Besley^4,32^ at vertical breakwaters, the research by Hughes^23^, Victor^25^ and Salauddin et al.^14,20^ for smooth sloping structures, and that by Zanuttigh^26^, Nørgaard^33^, and Molines^19^ for rubble mound breakwaters. The two-parameter Weibull distribution has also been incorporated in the EurOtop^11^ overtopping manuals to describe the wave-by-wave distribution of overtopping volumes at sea defences. The two-parameter Weibull cumulative distribution function can be expressed as:

1 $$P_{v} = 1 - \exp \left[ { - \left( \frac{V}{a} \right)^{b} } \right]{ }$$

where,*V* is the overtopping volume per wave (m^3^ per m or l per m), *P*_*v*_ is the probability that an individual overtopping volume will not exceed *V*, and *a* and *b* are scale and shape parameter for Weibull distribution, respectively. The dimensional scale factor, *a*, has units of overtopping discharge per unit length that normalises the Weibull distribution. The shape parameter, *b*, is non-dimensional and defines the extreme tail of Weibull distribution.

When measured wave-by-wave overtopping volumes follow the Weibull distribution, the measured mean overtopping volumes in a wave sequence equal the average overtopping volumes that are theoretically predicted from the Weibull distribution. Accordingly, EurOtop^11^ recommended the empirical relationship Eq. (2) that relates *a* and *b* of the Weibull distribution:

2 $$a = \left( {\frac{1}{{\Gamma \left( {1 + \frac{1}{b}} \right)}}} \right){ }\left( {\frac{{qT_{m} }}{{P_{ov} }}} \right){ }$$

where$\Gamma$ is the mathematical gamma function, *q* is the mean overtopping discharge per metre width (m^3^/s/m or l/s/m) and $P_{ov}$ is the proportion of overtopping waves (*N*_*ow*_*/N*_*w*_).

In Eq. (2), the coefficient *a* is proportional to the mean overtopping volumes in a test sequence, accordingly, scaling the wave by wave overtopping volumes in the Weibull distribution function as shown Eq. (1). The coefficient $1/ \Gamma \left( {1 + 1/b} \right)$ in Eq. (2) can be re-written as a dimensionless scale factor, $a^{\prime}$, as follows:

3 $$a^{\prime} = \left( {\frac{1}{{\Gamma \left( {1 + \frac{1}{b}} \right)}}} \right){ }$$

Van der Meer and Janssen^15^ suggested a shape factor *b* of 0.75 for sloping coastal defences, and Franco et al.^10^ proposed a shape factor, also of 0.75, for the analysis of vertical breakwaters under non impulsive wave conditions. The value of the dimensionless scale factor, $a^{\prime}$, corresponding to a *b* value of 0.75 is 0.84.

Besley^4^ reported that the Weibull *b* value is dependent on both the nature of impacting waves (impulsive or non-impulsive) at structures and the incident wave steepness, defined as: $s_{0p} = 2{\uppi }H_{m0} /\left( {{\text{g}}T_{p}^{2} } \right)$ where, $s_{0p}$ is the deep-water incident wave steepness, $H_{m0}$ is the significant wave height, and $T_{p}$ is the peak wave period. Consequently, Besley^4^ suggested increasing the value of *b* as incident wave steepness for sea defences also increases (as in the case of vertical breakwaters and smooth sloping structures). Using Besley’s^4^ formulation, EurOtop^11^ proposed two empirical formulae for estimating Weibull *b* at vertical breakwaters under non-impulsive Eq. (4) and impulsive wave conditions Eq. (5):

4 $$b = \left\{ {\begin{array}{*{20}c} {0.66 \,for\, s_{0p} = 0.02} \\ {0.82 \,for\, s_{0p} = 0.04} \\ \end{array} } \right.{ }\,h_{t}^{2} /(H_{m0} L_{m - 1,0} ) > 0.23{ }$$

5 $$b = 0.85\, h_{t}^{2} /(H_{m0} L_{m - 1,0} ) \le 0.23{ }$$

where, ${s}_{0p}$ is the deep-water incident wave steepness, ${H}_{m0}$ is the significant wave height, ${h}_{t}$ is the toe water depth and ${L}_{m-\mathrm{1,0}}$ is the deep-water wavelength based on ${T}_{m-\mathrm{1,0}}$.

Bruce et al.^13^ conducted a comprehensive two-dimensional physical modelling study on overtopping characteristics at rubble mound breakwaters, armoured with different armour units. The research concluded that the incident deep-water wave steepnesses had no influence on Weibull *b* for the conditions studied. A later study by Victor et al.^25^ at steep low crested sloping structures found that slope angle and relative crest freeboard had a strong influence on the Weibull *b* values but reported only a minimal influence of incident wave steepness on Weibull shape factors. In their work, Victor et al.^25^ proposed the following empirical formula to estimate the shape factor as a function of relative crest freeboard and seaward slope:

6 $$b = \exp \left[ { - 2.0\frac{{R_{c} }}{{H_{m0} }}} \right] + (0.56 + 0.15\cot \alpha ){ }$$

For vertical breakwaters with relatively high crest freeboard subjected to non-impulsive wave attack, Eq. (6) gives a shape factor *b* = 0.56.

For smooth sloping structures, Hughes et al.^23^ demonstrated that the best-fit to the extreme tail of the Weibull distribution can be obtained by considering the highest 10% of the individual overtopping volumes in the distribution. To estimate the Weibull *b* values at smooth sloping structures, the following correlation between shape factor *b* and the relative freeboard of the structure was established:

7 $${\text{b}} = \left[ {{\text{exp}}\left( { - 0.6\frac{{R_{c} }}{{H_{m0} }}} \right)} \right]^{1.8} + 0.64\;{\text{\,valid}}\;{\text{for}} - 2 < \frac{{R_{c} }}{{H_{m0} }} < 4.0$$

Zanuttigh et al.^26^ demonstrated that Weibull *b* for smooth and rubble mound structures can be better predicted by using the relative overtopping discharge *q/(*g*H*_*m0*_*T*_*m-1,0*_*)*, rather than relative freeboard (*R*_*c*_*/H*_*m0*_) and proposed two empirical formulae for estimating Weibull *b* as a function of this relative overtopping discharge (included later in EurOtop^9^) that are applicable to smooth Eq. (8) and rubble mound structures Eq. (9):

8 $${\text{b}} = 0.73 + 55\left( {\frac{q}{{{\text{g}}H_{m0} T_{m - 1,0} }}} \right)^{0.8}$$

9 $${\text{b}} = 0.85 + 1500\left( {\frac{q}{{{\text{g}}H_{m0} T_{m - 1,0} }}} \right)^{1.3}$$

For rock armoured rubble mound breakwaters, Nørgaard et al.^33^ proposed empirical relationships Eqns. (10) and (11) to estimate Weibull *b* values as a function of spectral significant wave height (*H*_*m0*_) and mean wave height (*H*_*1/10*_) of the top 10% highest waves, under depth-limited wave breaking conditions:

10 $${\text{b}} = 0.75{\text{ \,for\, }}H_{m0} /H_{\frac{1}{10}} { } \le 0.848\;and\;H_{m0} /h{ } \le 0.2$$

11 $${\text{b}} = - 6.1 + 8.08{ }\frac{{H_{m0} }}{{H_{\frac{1}{10}} }}\;\,for\;H_{m0} /H_{\frac{1}{10}} { } > 0.848\;and\;H_{m0} /h{ } > 0.2$$

Through an experimental programme on overtopping characteristics at steep low crested smooth sloping structures and vertical walls, Gallach et al.^22^ extended the Weibull *b* prediction method in Eq. (6) (from Victor et al.^25^), proposing the following relationship in terms of the structure slope and relative crest freeboard:

12 $$b = (0.59 + 0.23\cot \alpha ){\text{exp }}\left( { - 2.2\frac{{R_{c} }}{{H_{m0} }}} \right) + 0.83{ }$$

More recently, Molines et al.^19^ revisited existing empirical methods to estimate individual overtopping volumes for conventional mound breakwaters with relatively low overtopping volumes and with a low proportion of overtopping waves, proposing new empirical formulations to fit the 2-parameter Weibull distribution for individual overtopping volumes on mound breakwaters with low numbers of overtopping waves (*P*_*ow*_ < 0.2).

For a known number of overtopping waves (*N*_*ow*_), Weibull scale (*a*) and shape factors (*b*) in a test sequence, the maximum individual wave overtopping volume (*V*_*max*_) can be predicted using the following logarithmic function proposed by Besley^4^ in terms of *N*_*ow*_, *a*, and *b*:

13 $$V_{max} = a\left( {\ln N_{ow} } \right)^{1/b}$$

where$V_{max}$ is the maximum overtopping volume per meter structure width (m^3^ per m or l per m), and *N*_*ow*_ is the number of overtopping waves.

To estimate the proportion of overtopping waves (*P*_*ov*_) at plain vertical breakwaters, EurOtop^11^ recommends the following empirical formulations for non-impulsive Eq. (14) and impulsive conditions Eq. (15) wave conditions:

14 $$P_{ov} = \frac{{N_{ow} }}{{N_{w} }} = \exp \left[ { - 1.21\left( {\frac{{R_{c} }}{{H_{m0} }}} \right)^{2} } \right]$$

15 $$P_{ov} = \frac{{N_{ow} }}{{N_{w} }} = 0.024\left[ {\frac{{h_{t}^{2} }}{{(H_{m0} L_{m - 1,0} )}}\left( {\frac{{R_{c} }}{{H_{m0} }}} \right)} \right]^{ - 1} {\text{with a minimum estimated by Eq}}{. (14) }$$

Recent research has advanced our understanding of the distribution of individual wave overtopping volumes such that Weibull distribution shape factors can be reasonably well predicted as a function of wave characterises and wave overtopping discharges for sloping and rubble mound structures. However, to date, limited research has focussed specifically on improving the prediction tools for Weibull *b* at vertical coastal structures and methods used when designing coastal infrastructures are typically those that are specific to sloping structures. Uncertainties exist therefore, in estimating maximum overtopping volumes at these vertical structures and there remains an impetus for further research to address this knowledge deficit.

## Laboratory data

**Figure 1:**
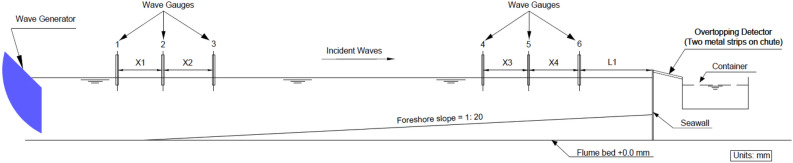
Schematic of the experimental test set-up for Warwick dataset. X1-X4 denotes the spacing of wave gauges and L1 indicates the position of wave gauges from the seawalls in the laboratory set-up (see Ref. ^3,6,30^).

The data and analyses presented in this paper is underpinned by a large dataset collated from studies conducted in two research grade laboratory wave flumes. The data comprises measurements of wave-by-wave overtopping volumes at plain vertical seawalls for four test series covering the range of hydrodynamic and structural configurations described in Table 1. Three test series adopted for the analysis are conducted at the University of Warwick, on plain vertical seawalls. Test Series 1 presented the recent undertaken as part of the EU Interreg Ecostructure project on an impermeable sloping foreshore (1 V:20H) [3, 36]; Series 2 investigated overtopping volumes with and without retrofitting configurations^6^; Series 3 assessed overtopping volumes with permeable (shingle) and impermeable sloping foreshore (1 V:20H)^30^. In Fig. 1, a schematic of the experimental set up for Test Series 1 -3 are presented. To complement the datasets, Test Series 4 includes the experiments conducted on plain vertical seawalls as part of the UK EPSRC funded Violent Overtopping by Waves at Seawalls (VOWS) project^28,35^. Weibull b values collected from the VOWS project (Test Series 4 in this study) are analyzed in the present study for the first time, to examine the applicability of the existing formulae in the prediction of Weibull shape factor for vertical seawalls. The differences in datasets are provided in Table 1 with references to the detailed laboratory experiments of each dataset.

**Table 1:** Ranges of wave and structural configurations correspond to dataset considered within this study.

Dataset	Minimum		Parameter		Maximum
Test series 1	1.20	≤	Relative Freeboard, R_c_/H_m0_	≤	2.86
	0.018	≤	Wave Steepness, s_0p_	≤	0.06
	0.01840	<	Impulsive Parameter, ${{h}_{t}}^{2}/{(H}_{m0}{L}_{m-\mathrm{1,0}})$,		0.63199
	1 (V): 20 (H) = Slope of the foreshore				
Test Series 2	0.86	≤	Relative Freeboard, R_c_/H_m0_	≤	3.87
	0.017	≤	Wave Steepness, s_0p_	≤	0.054
	0.0146	<	Impulsive Parameter, ${{h}_{t}}^{2}/{(H}_{m0}{L}_{m-\mathrm{1,0}})$,		0.9775
	1 (V): 20 (H) = Slope of the foreshore				
Test Series 3	0.80	≤	Relative Freeboard, R_c_/H_m0_	≤	4.88
	0.015	≤	Wave Steepness, s_0p_	≤	0.06
	0.017	<	Impulsive Parameter, ${{h}_{t}}^{2}/{(H}_{m0}{L}_{m-\mathrm{1,0}})$,		1.027
	1 (V): 20 (H) = Slope of the foreshore				
Test Series 4	1.22	≤	Relative Freeboard, R_c_/H_m0_	≤	3.77
	0.004	≤	Wave Steepness, s_0p_	≤	0.043
	0.025	<	Impulsive Parameter, ${{h}_{t}}^{2}/{(H}_{m0}{L}_{m-\mathrm{1,0}})$,		0.550
	1 (V): 10 (H) = Slope of the foreshore

For both Warwick and VOWS datasets, wave-by-wave overtopping volumes were collected by using an overtopping container suspended from a loadcell and an overtopping detector positioned along the crest of the seawalls see ^3,6,28,30^. Tests were performed using both impulsive and non-impulsive wave conditions, with an impermeable plain vertical seawall. A minimum of 1000 JONSWAP pseudo random waves was performed per test case. Details of the overtopping measurement and analysis techniques are reported in respective studies see ^3,6,28,30^. The resulting distribution of individual wave overtopping volumes followed a 2-parameter Weibull distribution as reported in those studies ^3,6,28,30^, which then further processed to evaluate the Weibull *b* or shape factor of the distribution for each test run.

## Results and discussion

### Weibull plot of individual overtopping wave volumes

**Figure 2:**
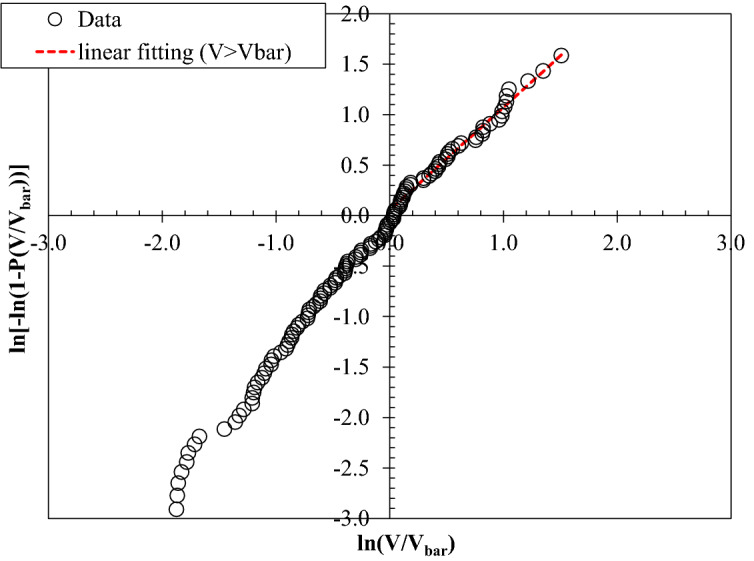
Weibull plot of overtopping volumes for an experiment representing a typical low wave steepness condition, s_0p_ = 1.8% with a significant wave height of 0.07 m.

**Figure 3:**
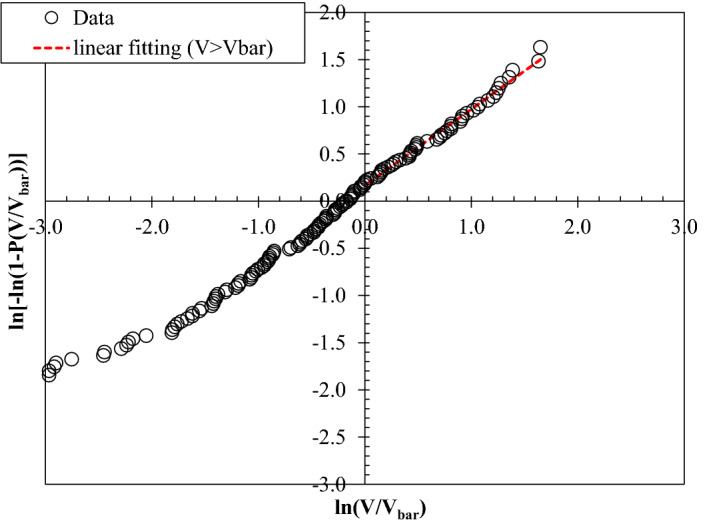
Weibull plot of overtopping volumes for an experiment representing a typical storm wave condition, s_op_ = 5% with a significant wave height of 0.08 m.

The measured wave by wave overtopping volumes are plotted on a Weibull scale for each experiment to determine the distribution of these volumes. Figures 2 and 3 illustrate the distributions of measured wave-by-wave volumes at plain vertical walls with three tested bed configurations, subjected to the same incident wave condition, where *V* denotes the individual overtopping volume, *P*(*V*) refers to the probability of exceedance, and *V*_*bar*_ represents the mean overtopping volume. The straight line on the Weibull plot signifies that the measured data points follow the two parameter Weibull distribution for *V* > *V*_*bar*_. Figure 2 displays the data points that correspond to storm wave condition (s_m-1,0_ = 6%), whereas Fig. 3 portrays the wave attack with relatively low wave steepness (s_m-1,0_ = 1.5%) at plain vertical walls.

Overall, a linear trend of data points can be observed from Figs. 2 and 3 for both low and high wave steepness, indicating that the measured individual overtopping volumes fit the two-parameter Weibull distribution for the tested conditions.

In the Weibull distribution of wave-by-wave volumes, the distributions of small overtopping volumes (lower part) in many cases deviated from the inclination of the upper part of the distribution^25,26,36^. Many researchers have reported that higher wave-by-wave volumes give a good fit to Weibull distribution and offer reliable estimation of extreme individual overtopping wave volumes^4,15^. Generally, practitioners mainly focus on the largest wave overtopping volumes, wherein the upper part of the distribution is used to get a good fit at the extreme overtopping wave volumes. By adopting the procedure prescribed by Pearson et al.^34^, the best-fit linear trend lines in Figs. 2 and 3 are plotted by considering the upper part of the distribution of wave-by-wave volumes, i.e. considering overtopping volumes greater than the average values (*V* > *V*_*bar*_), as indicated in the graphs.

The Weibull *b* parameter can be determined from the inclination of the best-fitting line. From the resulting Weibull distribution of overtopping volumes, the shape factor *b* of the distribution was determined for each test.

### Influence of probability of overtopping waves on Weibull shape parameter

**Figure 4:**
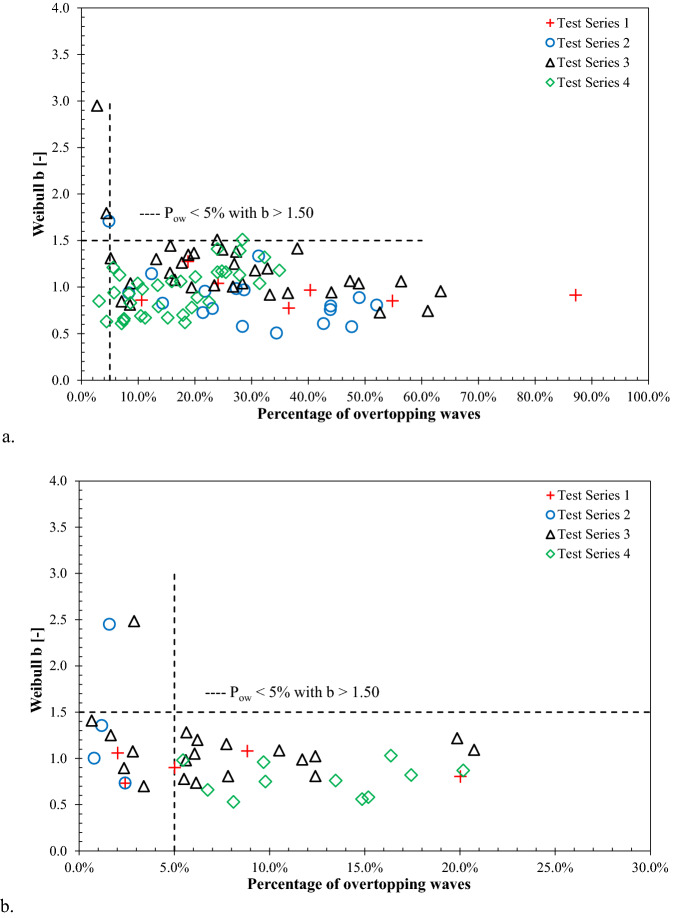
Influence of overtopping waves probability on the Weibull *b* parameters: **(a)** impulsive wave conditions, and **(b)** non-impulsive wave conditions.

The variation of Weibull shape parameter *b* with the percentage of overtopping waves is displayed in Fig. 4, subjected to both impulsive and non-impulsive conditions. The results show that the measured Weibull *b* values fell within the range of 0.65–1.50 for most of the tested conditions. However, in some cases, higher values of (*b* > 1.5) Weibull shape parameter was recorded (Fig. 4).

It is noted that the tests with very low overtopping waves resulted in higher Weibull shape parameter (b > 1.5). Similar characteristics of Weibull shape parameter with respect to low overtopping waves (below 5% of overtopping waves) were also reported by Zanuttigh et al.^26^ for rubble mound breakwaters.

### Influence of wave steepness on Weibull b

**Figure 5:**
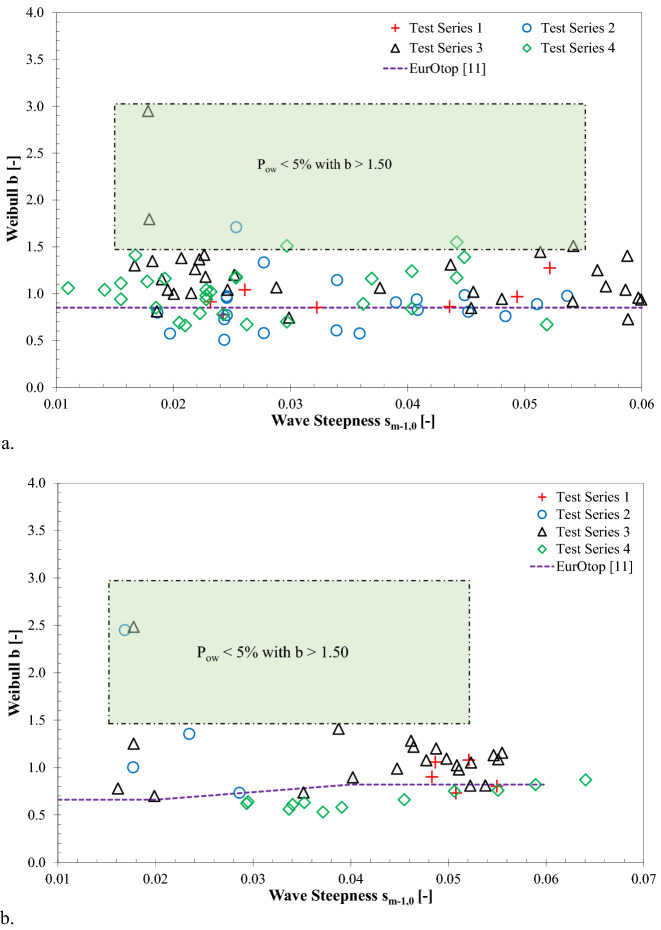
Influence of incident wave steepness on Weibull *b* parameters: **(a)** impulsive wave conditions,and **(b)** non-impulsive wave conditions.

To investigate the influence of wave steepness on the Weibull distribution of the individual volumes, the measured shape factor *b* was plotted as a function of wave steepness, s_m-1,0_, for both impulsive and non-impulsive conditions (see Fig. 5). The solid lines in Fig. 5 represent the *b* values (*b* = 0.66 for s_m-1,0_ = 0.02, and *b* = 0.82 for s_m-1,0_ = 0.04 under non-impulsive conditions, while *b* = 0.85 for all s_m-1,0_ under impulsive conditions), as recommended by Besley^4^, which have been incorportaed in the new overtopping manual^11^.

Figure 5 highlights scatter in the data points, signifying no clear influence of wave steepness on the shape of the Weibull distribution. It was observed that the measured values of the shape parameter were higher than the values suggested by^11^ for both impulsive and non-impulsive wave conditions.

### Influence of impulsiveness parameter on Weibull b

**Figure 6:**
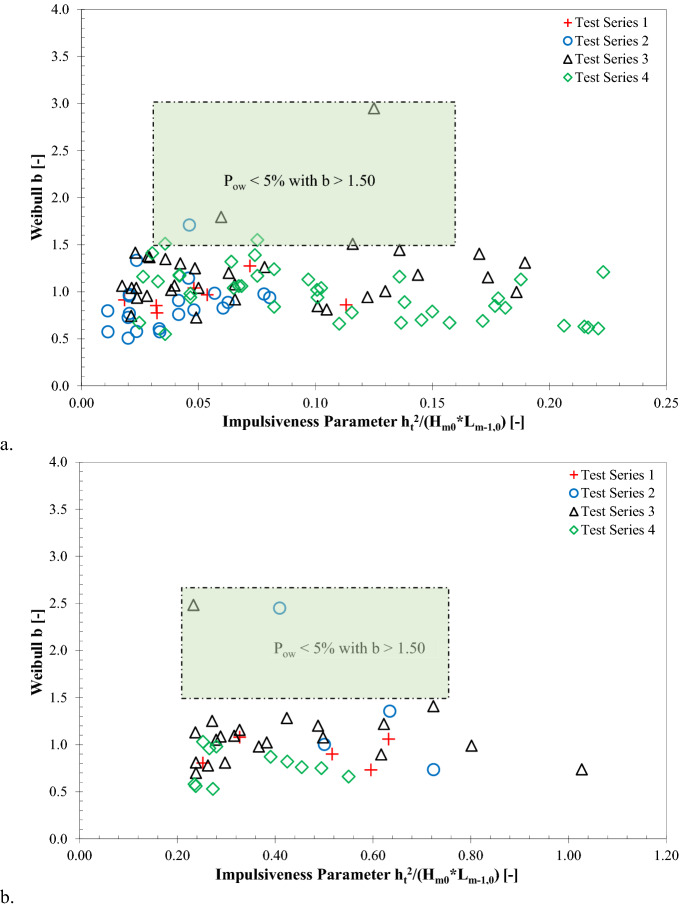
Influence of impulsiveness parameter on Weibull *b* parameters: **(a)** impulsive wave conditions and **(b)** non-impulsive wave conditions.

In Fig. 6, the Weibull *b* values are plotted as a function of impulsiveness parameter, ${{h}_{t}}^{2}/{(H}_{m0}{L}_{m-\mathrm{1,0}})$, where *h*_*t*_ refers to the toe water depth, *H*_*m0*_ indicates the significant wave height at the structure, and *L*_*m-1,0*_ represents the deep-water wavelength. The results show scattering, thus indicating that the Weibull *b* values are not influenced by the variation of wave impulsiveness parameter for the tested impulsive and non-impulsive conditions.

### Influence of relative local water depth on Weibull b

**Figure 7:**
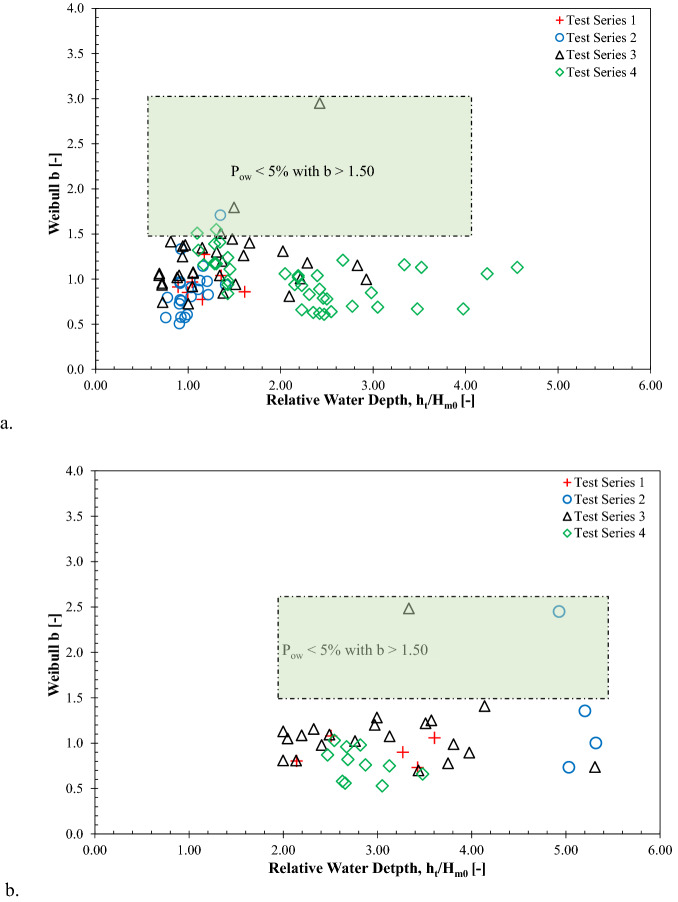
Influence of relative local water depth on Weibull *b* parameters: **(a)** impulsive wave conditions and **(b)** non-impulsive wave conditions.

Figure 7 illustrates the effect of the relative toe water depth (*h*_*t*_*/H*_*m0*_) on the observed Weibull *b* values for both impulsive and non-impulsive wave conditions. The observed Weibull *b* values did not vary with the variation of relative toe water depths for all considered configurations within this study. Hence, it can be concluded that, for the conditions tested, there is no apparent influence of the relative toe water depths on the shape factor.

### Influence of relative crest freeboard on Weibull b

The analysis of the distribution of wave-by-wave overtopping volumes reveal an influence of relative crest freeboard on the Weibull shape factor for rubble mound and smooth sloping structures (see^23,25^). In order to estimate the Weibull *b* values at smooth sloping structures, Hughes et al.^23^ established a correlation between shape factor and relative freeboard see Eq. (7). For relatively steep low crested sloping structures, Victor et al.^25^ prescribed an empirical formula see Eq. (6) to estimate the shape factor as a function of relative freeboard and seaward slope. For vertical walls with relatively high freeboard under non-impulsive conditions, Eq. (6) yield into *b* = 0.56.

**Figure 8:**
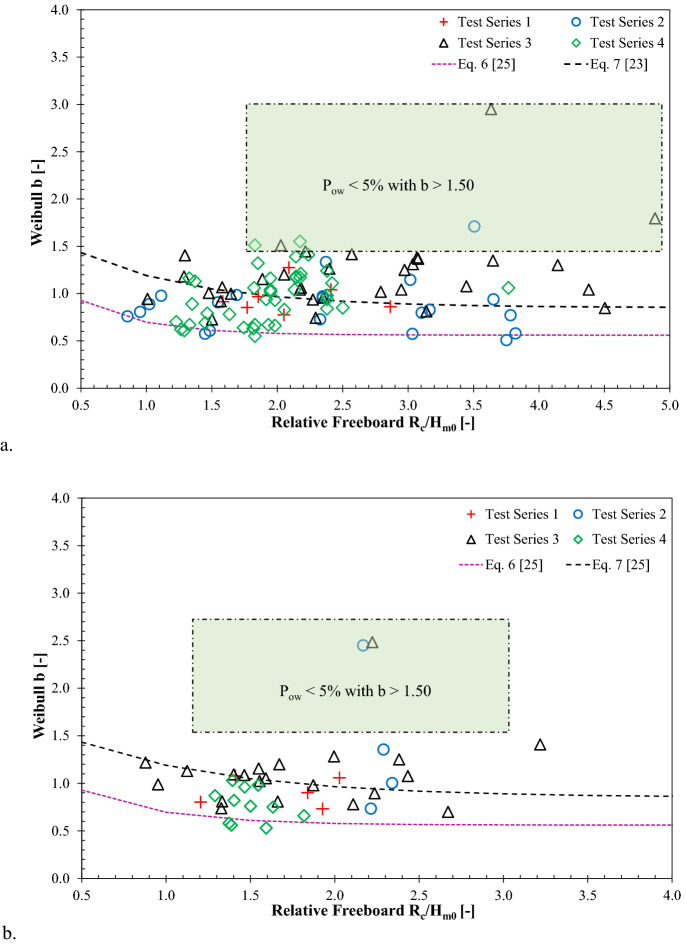
Influence of relative crest freeboard on Weibull *b* parameters: **(a)** impulsive wave conditions and **(b)** non-impulsive wave conditions.

Figure 8 portrays the effect of relative crest freeboard on the Weibull shape factor under both impulsive and non-impulsive conditions. The data retrieved from the VOWS project are also presented in Fig. 8. The existing empirical predictions for smooth sloping (E. 6) and rubble mound Eq. (7) structures are displayed in Fig. 8 to examine the implications of these formulae for vertical walls.

For both impulsive and non-impulsive wave attack, it is evident from the graphs that the data fairly adhered to the general trend of the prediction line, as reported by^23^ for smooth sloping structures, particularly given the varied structural configurations. As mentioned previously, the exception is that some data were below 5% of the overtopping waves. The measured Weibull *b* values for plain vertical walls did not perfectly fit the predictions of^25^, subjected to impulsive and non-impulsive conditions. Nevertheless, based on the predictions reported by^25^, Eq. (6), can not provide appropriate predictions for impulsive wave attack at plain vertical walls.

### Influence of relative overtopping discharge on Weibull b

**Figure 9:**
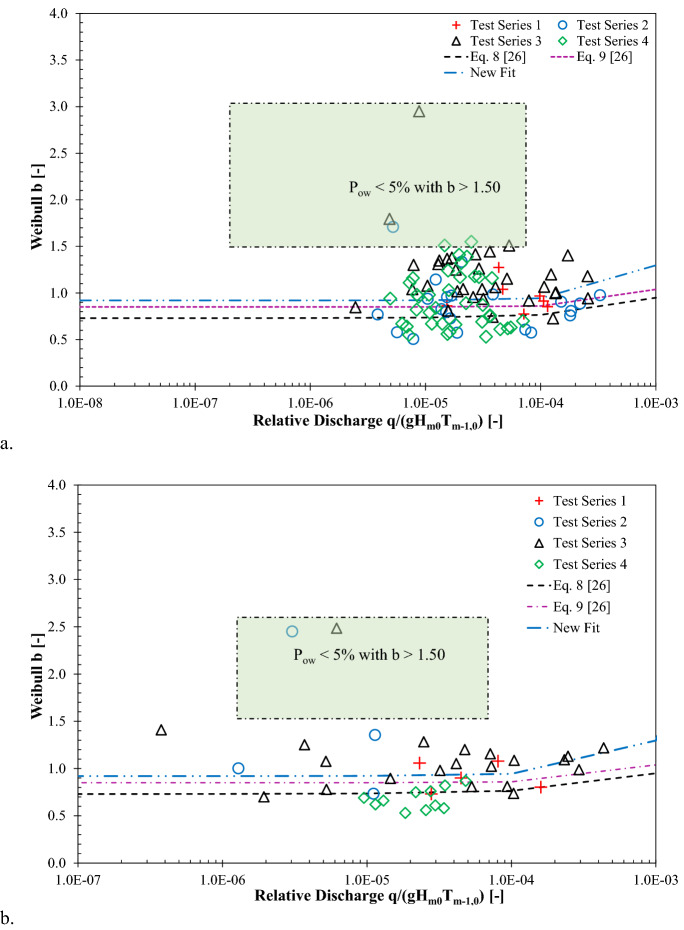
Influence of relative overtopping discharge on Weibull *b* parameters: **(a)** impulsive wave conditions and **(b)** non-impulsive wave conditions.

Figure 9 analyses the measured Weibull shape parameter as a function of relative discharge *q/*(g*H*_*m0*_*T*_*m-1,0*_). The Weibull *b* values for the vertical walls tested in VOWS project are presented in Fig. 9. The dashed lines signify the empirical predictions made by Zanuttigh et al.^26^ for smooth and rubble mound structures.

For impulsive wave conditions, the measured Weibull *b* values are slightly higher than that of empirical predictions see Eq. (8) reported by^26^. Figure 9 (a) highlights the conservative approach of Eq. (8) for prediction of the shape parameter for vertical breakwaters. Upon adopting the methodology prescribed by^26^, a new and improved trend for plain vertical walls is proposed in this study using the data retrieved from measured Weibull *b* values and the VOWS project see Eq. (16). The scatter data points that corresponded to low overtopping waves (below 5%) and higher Weibull *b* values were excluded from Eq. (16).

16 $${\text{b}} = 0.92 + 1500\left( {\frac{q}{{{\text{g}}H_{m0} T_{m - 1,0} }}} \right)^{1.3} {\text{ valid for }}0.80 \le \frac{{R_{c} }}{{H_{m0} }} \le 4.88$$

### Prediction of Weibull b values at plain vertical walls

In order to estimate Weibull *b* at vertical walls with gravel foreshores, to date, there is relatively limited prediction guidance available in the literature. Considering an impermeable foreshore slope, EurOtop^11^ reported *b* = 0.85 for impulsive conditions, while *b* = 0.66 for wave steepness (s_m-1,0_) of 0.02, and *b* = 0.82 for s_m-1,0_ = 0.04 under non-impulsive conditions. As evident from this study (see Fig. 5), no influence of wave steepness was noted on the reported Weibull *b* values for both impulsive and non-impulsive wave conditions.

In revising the prediction tools of Weibull *b* values at vertical walls, the test results were compared with the appropriate predictions reported in the literature (see Figs. 5, 8, and 9). In order to quantify the reliability of the prediction formulae, statistical error indicators, including scatter index (SI), Bias, and root mean square error (RMSE), were determined using the measured and estimated Weibull *b* values (see Eqns. (17)–(19)). The SI measure signifies the relative scatter of the measured data points, while the Bias value represents the difference between the measured and estimated mean value, and the RMSE values are determined to quantify the accuracy of the prediction formulae.

17 $$SI = \frac{1}{{\overline{\left| X \right|} }}\sqrt {\frac{1}{{N_{test} }} \mathop \sum \limits_{n = 1}^{{N_{test} }} \left[ {\left( {b_{estimated} } \right)_{n} - \left( {b_{measured} } \right)_{n} } \right]^{2} } *100$$

18 $$Bias = \frac{1}{{N_{test} }} \mathop \sum \limits_{n = 1}^{{N_{test} }} \left[ {\left( {b_{estimated} } \right)_{n} - \left( {b_{measured} } \right)_{n} } \right]$$

19 $$RMSE = \sqrt {\frac{1}{{N_{test} }} \mathop \sum \limits_{n = 1}^{{N_{test} }} \left[ {\left( {b_{measured} } \right)_{n} - \left( {b_{estimated} } \right)_{n} } \right]^{2} }$$

where,*N*_*test*_ denotes the number of experimental data, *b*_*measured*_ and *b*_*estimated*_ are the measured and estimated Weibull b values respectively, and $\overline{X}$ denotes the average of *b*_*measured*_ values.

For all the test configurations subjected to impulsive wave attack considered in this study, the error measures of the empirical formulae are presented in Table 2. As noted in Fig. 8(a) and Fig. 9(a), it is evident that Weibull *b* values did not adhere to the predictions formulae proposed by^25^ (see Eq. (6)) and^26^ (see Eq. (9)) for sloping structures, thus excluded from Table 2.

**Table 2:** Summary of the statistical indicators for empirical formulae in predicting Weibull *b* values at plain vertical walls under impulsive conditions.

Error Indicators	Dataset	Hughes et al. ^23^—Eq. (7)	Zanuttigh et al. ^26^—Eq. (8)	This study (Eq. (16))
SI (%)	Test Series 1	17.06	19.12	16.42
	Test Series 2	37.74	31.31	28.49
	Test Series 3	21.72	25.02	20.78
	Test Series 4	29.04	29.12	26.54
BIAS	Test Series 1	0.01	− 0.10	− 0.02
	Test Series 2	0.11	− 0.02	0.06
	Test Series 3	− 0.14	− 0.22	− 0.15
	Test Series 4	0.003	− 0.13	− 0.06
RMSE	Test Series 1	0.16	0.18	0.16
	Test Series 2	0.33	0.27	0.25
	Test Series 3	0.26	0.30	0.25
	Test Series 4	0.29	0.29	0.26

Table 2 shows that the SI of the improved predictive relation proposed in this study (i.e., Eq. (16)) are lower than those reported by^23^ and^26^, indicating that the estimated values of the proposed formula are less scattered than the those determined from the literature. The Bias values tabulated in Table 1 show the similarity between the average absolute error by^23^ and the new formulae, although the latter appear to be relatively lower than those reported by^26^. The negative Bias values in Table 2 show that the prediction formulae provided underestimation of Weibull *b* values. Table 2 shows that for all the configurations examined in this study, the observed RMSE values of the proposed equation Eq. (16) are relatively lower than those reported in the literature; signifying that the proposed new formula offers a good fit with the measurements.

For the tested non-impulsive wave conditions, the error measures of the empirical formulae in predicting the Weibull *b* values at plain vertical walls are shown in Table 3. The results show that both existing (Eqns. (7)–(8)) and the new proposed formulae succeeded in providing reliable predictions for shape parameter *b*, which is demonstrated by providing reasonably lower SI, Bias, and RMSE values for all the configurations. Table 3 also shows that the measured *b* values did not follow the predictions of those reported by^25^ (see Eq. (6)) for vertical walls under non-impulsive wave attack.

**Table 3:** Summary of the statistical indicators for empirical formulae in predicting Weibull *b* values at plain vertical walls under non-impulsive conditions.

Error Indicators	Dataset	Hughes et al. ^23^—Eq. (7)	Victor et al. ^25^—Eq. (6)	Zanuttigh et al. ^26^—Eq. (8)	This study (Eq. (16))
SI (%)	Test Series 1	20.62	37.50	16.42	15.55
	Test Series 2	56.93	75.31	60.95	57.87
	Test Series 3	24.30	40.68	20.81	16.92
	Test Series 4	41.93	29.81	24.00	29.26
Bias	Test Series 1	0.11	− 0.31	− 0.06	0.02
	Test Series 2	− 0.45	− 0.81	− 0.53	− 0.46
	Test Series 3	0.03	− 0.29	− 0.14	− 0.07
	Test Series 4	0.28	− 0.16	0.08	0.15
RMSE	Test Series 1	0.19	0.34	0.15	0.14
	Test Series 2	0.79	1.04	0.84	0.80
	Test Series 3	0.27	0.45	0.23	0.19
	Test Series 4	0.32	0.23	0.19	0.23

Following the statistical analysis of error indexes for both existing and the proposed new formulae, this study provides an enhanced guidance for the estimation of Weibull *b* values at plain vertical walls subject to both impulsive and non-impulsive wave conditions (see Table 4).

**Table 4:** Guidance for the estimation of Weibull *b* values at vertical breakwaters.

Wave conditions	Using R_c_/H_m0_	Using q/(gH_m0_T_m-1,0_)	Validity range
Impulsive	Not applicable	Equation (16)	0.80 ≤ R_c_/H_m0_ ≤ 4.88
Non-Impulsive	Equation (7)	Equation (8) and/or (16)	0.80 ≤ R_c_/H_m0_ ≤ 4.88

## Conclusions

Data and information regarding the distribution of individual wave overtopping volumes and maximum wave by wave overtopping volume (*V*_*max*_) in a sequence of overtopping events is key for the reliable prediction of overtopping hazards and profiling the associated risks along defence lines. The 2-parameter Weibull function is typically employed to illustrate the distribution of wave by wave overtopping volumes and derive the maximum individual overtopping volume. An accurate prediction of Weibull shape factor (*b*) generally leads to a more precise estimation of *V*_*max*_. This study aims to investigate the Weibull distribution of wave overtopping volumes and assess the performance of existing prediction methods in deriving Weibull *b* values at vertical seawalls. Wave-by-wave overtopping volume measurements on plain vertical seawalls collected from small scale physical model experiments^6,28,30^ are examined and compared to the existing prediction methods.

The variation of measured Weibull *b* values with respect to various wave conditions and structural parameters (i.e., probability of overtopping waves, wave steepness, impulsiveness parameter, local water depth, crest freeboard, and overtopping discharge) are assessed. Measurements of Weibull *b* values for both impulsive and non-impulsive wave conditions, showed that the proportion of waves that overtopped the crest of seawalls has a strong influence on the distribution of wave overtopping volumes, i.e., Weibull shape parameter. It was found that for vertical seawall tests with relatively low probability of overtopping waves resulted comparatively higher values of (*b* > 1.5) Weibull shape parameters, similar to the case for smooth and rubble mound structures reported by Zanuttigh et al.^26^. The results of this study conclude that the influence of incident wave steepness is limited on the Weibull distribution of wave-by-wave overtopping volumes on a plain vertical wall. When the observed Weibull *b* values for the conditions covered within this study are compared with the existing prediction methods as suggested by Besely^4^ and later incorporated in EurOtop^11^ which are based on incident wave steepness, it was found that these empirical formulae are unable to estimate the Weibull *b* values at plain vertical walls. Deviations of observed Weibull *b* values from Besely^4^ predictions have also been previously reported in the literature [e.g., ^20,22,25^].

For both impulsive and non-impulsive conditions, effects of impulsiveness parameter and relative local water depth on the distribution of wave-by-wave overtopping volumes at plain vertical walls are not clearly apparent. The study findings however suggest that the crest freeboard has a strong influence on the estimation of Weibull *b* parameters for plain vertical seawalls. The findings of this work in regard to the relationship between Weibull *b* values and relative crest freeboard are in line with the findings of previous studies on sloping dikes (see^21,23^) and rubble mound breakwaters (see^19^). Weibull *b* values reported in this work under non-impulsive wave conditions at seawalls were shown to be somewhat consistent with empirical predictions using relative crest freeboard as reported for sloping structures (Eq. (7)) and vertical walls Eq. (6). Statistical error analysis shows that measurements of Weibull *b* values considered within this study have overall good agreement with the existing relationship for sloping structures (i.e., Eq. (7)), when compared to the trendline of Eq. (6) for vertical walls. It is therefore recommended that adopting prediction methods of sloping walls (i.e., Eq. (7), with a new validity range, see Table 4) can be employed in estimating Weibull *b* values at plain vertical seawalls under non-impulsive conditions.

Recent advancements in estimation of Weibull *b* parameters at sea defences (see for instance, Eqns. (8) and (9) for smooth and rubble mound structures, respectively) showed that there is a strong correlation between the relative wave overtopping discharge and Weibull *b* values. Given the measurements of Weibull *b* values of this study were overall consistent with the existing empirical relationship of overtopping discharge and Weibull *b* values^26^, despite of different structural configurations, the empirical formulae (adopting Eq. (8) with new validity range) can be also applied to estimate Weibull *b* values at vertical breakwaters, particularly for non-impulsive wave conditions. To improve the accuracy of estimation of Weibull *b* for vertical walls under impulsive and non-impulsive wave attacks, a new formula Eq. (16) is proposed in this study with detailed error statistics analysis.

Prior to this study, there was no attempt to assess the influence of overtopping discharge on the estimation of Weibull *b* values for vertical breakwaters. This study proposed a new set of formulae for the prediction of Weibull *b* values at seawalls using the crest freeboard and/or relative overtopping rates, subjected to both impulsive and non-impulsive conditions (Table 4). The findings of this research will be of interest to engineers, practitioners, and managers in deriving a reliable prediction of overtopping volumes and subsequently in profiling the associated hazards along the coastal defences.

## Data Availability

The data that support the findings of this study are available from the corresponding author upon reasonable request.

## References

[1] Allen, M., Antwi-Agyei, P., Aragon-Durand, F., Babiker, M., Bertoldi, P., Bind, M., Brown, S., Buckeridge, M., Camilloni, I., and Cartwright, A., Technical Summary: Global warming of 1.5° C. An IPCC Special Report on the impacts of global warming of 1.5° C above pre-industrial levels and related global greenhouse gas emission pathways, in the context of strengthening the global response to the threat of climate change, sustainable development, and efforts to eradicate poverty., in Intergovernmental Panel on Climate Change. 2019: Geneve, Switzerland.

[2] Vitousek S, Barnard PL, Fletcher CH, Frazer N, Erikson L, Storlazzi CD (2017). Doubling of coastal flooding frequency within decades due to sea-level rise. Sci. Rep..

[3] O'Sullivan, J.J., Salauddin, M., Abolfathi, S., and Pearson, J.M., Effectiveness of eco-retrofits in reducing wave overtopping on seawalls. Coastal Engineering Proceedings, 2020(36v): p. 13-13

[4] Besley, P., Overtopping of seawalls—design and assessment manual. In R&D Technical Report W 178. Environment Agency: UK. (1999)

[5] Salauddin, M., O'Sullivan, J.J., Abolfathi, S., and Pearson, J.M. Extreme wave overtopping at ecologically modified sea defences. In EGU General Assembly Conference Abstracts. (2020).

[6] Dong S, Abolfathi S, Salauddin M, Tan ZH, Pearson JM (2020). Enhancing climate resilience of vertical seawall with retrofitting-A physical modelling study. Appl. Ocean Res..

[7] Salauddin M, Pearson JM (2018). A laboratory study on wave overtopping at vertical seawalls with a shingle foreshore. Coast. Eng. Proc..

[8] Bruce T, Van der Meer JW, Pullen T, Allsop W (2010). Wave overtopping at vertical and steep structures. Handbook of coastal and ocean engineering.

[9] Dong, S., Salauddin, M., Abolfathi, S., Tan, Z.H., and Pearson, J.M. The influence of geometrical shape changes on wave overtopping: a laboratory and SPH numerical study, in Coasts, Marine Structures and Breakwaters 2017: Realising the Potential. ICE Publishing. p. 1217–1226. (2018)

[10] Franco L, De Gerloni M (1995). and Van der Meer, J., *Wave overtopping on vertical and composite breakwaters*. Coast. Eng..

[11] EurOtop, Manual on wave overtopping of sea defences and related structures. , in 2nd Edition. (2018): www.overtopping-manual.com.

[12] Salauddin M, Broere A, Van der Meer JW, Verhagen HJ, Bijl E (2017). First Tests on the Symmetrical Breakwater Armor Unit Crablock. Coast. Eng. J..

[13] Bruce T, Van der Meer JW, Franco L, Pearson JM (2009). Overtopping performance of different armour units for rubble mound breakwaters. Coast. Eng..

[14] Salauddin M, Pearson JM (2020). Laboratory investigation of overtopping at a sloping structure with permeable shingle foreshore. Ocean Eng..

[15] Van der Meer, J.W. and Janssen, J.P.F.M., Wave run-up and wave overtopping at dikes, in Delft Hydraulics No. 485. 1994. p. 22.

[16] Van der Meer JW, Bruce T (2014). New physical insights and design formulas on wave overtopping at sloping and vertical structures. J. Waterw. Port Coast. Ocean Eng..

[17] Etemad-Shahidi A, Jafari E (2014). New formulae for prediction of wave overtopping at inclined structures with smooth impermeable surface. Ocean Eng..

[18] Koosheh A, Etemad-Shahidi A, Cartwright N, Tomlinson R, van Gent MRA (2021). Individual wave overtopping at coastal structures: A critical review and the existing challenges. Appl. Ocean Res..

[19] Molines J, Herrera MP, Gómez-Martín ME, Medina JR (2019). Distribution of individual wave overtopping volumes on mound breakwaters. Coast. Eng..

[20] Salauddin M, O'Sullivan JJ, Abolfathi S, Dong S, Pearson JM (2020). Distribution of individual wave overtopping volumes on a sloping structure with a permeable foreshore. Coast. Eng. Proc..

[21] Salauddin, M. and Pearson, J.M., Distribution of wave by wave overtopping volumes at vertical seawalls, in 3rd International Conference on Protection Against Overtopping. 2018, HR Wallingford: Lake District, UK.

[22] Gallach-Sánchez D, Troch P, Kortenhaus A (2018). Average and wave-by-wave overtopping performance of steep low-crested structures. Coast. Eng. Proc..

[23] Hughes SA, Thornton CI, Van der Meer JW, Scholl B (2012). Improvements in describing wave overtopping processes. Coast. Eng. Proc..

[24] Iuppa C, Cavallaro L, Musumeci RE, Vicinanza D, Foti E (2019). Empirical overtopping volume statistics at an OBREC. Coast. Eng..

[25] Victor L, Van der Meer J, Troch P (2012). Probability distribution of individual wave overtopping volumes for smooth impermeable steep slopes with low crest freeboards. Coast. Eng..

[26] Zanuttigh, B., Van der Meer, J.W., Bruce, T., and Hughes, S.A. Statistical characterisation of extreme overtopping wave volumes. In From Sea to Shore–Meeting the Challenges of the Sea: (Coasts, Marine Structures and Breakwaters 2013). 2013. ICE Publishing.

[27] Abolfathi S, Shudi D, Borzooei S, Yeganeh-Bakhtiari A, Pearson JM (2018). Application of smoothed particle hydrodynamics in evaluating the performance of coastal retrofit structures. Coast. Eng. Proc..

[28] Pearson, J.M., Bruce, T., and Allsop, W., Prediction of wave overtopping at steep seawalls—variabilities and uncertainties, in Ocean Wave Measurement and Analysis pp 1797–1808 (2001)

[29] Dong S, Abolfathi S, Salauddin M, Pearson JM (2020). Spatial distribution of wave-by-wave overtopping at vertical seawalls. Coast. Eng. Proc..

[30] Salauddin M, Pearson JM (2019). Wave overtopping and toe scouring at a plain vertical seawall with shingle foreshore: A Physical model study. Ocean Eng..

[31] Gaeta MG, Guerrero M, Formentin SM, Palma G, Zanuttigh B (2020). Non-intrusive measurements of wave-induced flow over dikes by means of a combined ultrasound doppler velocimetry and videography. Water.

[32] Salauddin, M., Peng, Z., and Pearson, J. (2021). The effects of wave impacts on toe scouring and overtopping concurrently for permeable shingle foreshores. EGU General Assembly 2021, online, 19–30 Apr 2021, EGU21-548

[33] Nørgaard JQH, Andersen TL, Burcharth HF (2014). Distribution of individual wave overtopping volumes in shallow water wave conditions. Coast. Eng..

[34] Pearson JM, Bruce T, Allsop W, Gironella X (2003). Violent wave overtopping–measurements at large and small scale. Coastal Engineering 2002: Solving Coastal Conundrums.

[35] Salauddin M, O'Sullivan JJ, Abolfathi S, Pearson JM (2021). Eco-engineering of seawalls—an opportunity for enhanced climate resilience from increased topographic complexity. Front. Mar. Sci..

[36] Peng Z, Zou Q.-P (2011). Spatial distribution of wave overtopping water behind coastal structures. Coastal
Engineering.

